# Glutamine starvation enhances PCV2 replication via the phosphorylation of p38 MAPK, as promoted by reducing glutathione levels

**DOI:** 10.1186/s13567-015-0168-1

**Published:** 2015-03-18

**Authors:** Xingxiang Chen, Xiuli Shi, Fang Gan, Da Huang, Kehe Huang

**Affiliations:** College of Veterinary Medicine, Nanjing Agricultural University, Nanjing, China; Department of Chemistry, Rice University, Houston, Texas 77005 USA

## Abstract

Glutamine has a positive effect on ameliorating reproductive failure caused by porcine circovirus type 2 (PCV2). However, the mechanism by which glutamine affects PCV2 replication remains unclear. This study was conducted to investigate the effects of glutamine on PCV2 replication and its underlying mechanisms in vitro. The results show that glutamine promoted PK-15 cell viability. Surprisingly, glutamine starvation significantly increased PCV2 replication. The promotion of PCV2 replication by glutamine starvation disappeared after fresh media with 4 mM glutamine was added. Likewise, promotion of PCV2 was observed after adding buthionine sulfoximine (BSO). Glutamine starvation or BSO treatment increased the level of p38 MAPK phosphorylation and PCV2 replication in PK-15 cells. Meanwhile, p38 MAPK phosphorylation and PCV2 replication significantly decreased in p38-knockdown PK-15 cells. Promotion of PCV2 replication caused by glutamine starvation could be blocked in p38-knockdown PK-15 cells. Therefore, glutamine starvation increased PCV2 replication by promoting p38 MAPK activation, which was associated with the down regulation of intracellular glutathione levels. Our findings may contribute toward interpreting the possible pathogenic mechanism of PCV2 and provide a theoretical reference for application of glutamine in controlling porcine circovirus-associated diseases.

## Introduction

Porcine circovirus type 2 (PCV2) has been associated with porcine circovirus-associated diseases (PCVD), which collectively include the post-weaning multi-systemic wasting syndrome (PMWS), the porcine dermatitis and nephropathy syndrome (PDNS), porcine respiratory and reproductive disorders, proliferative and necrotizing pneumonia (PNP), congenital tremors (CT), and enteric disease [1]. Infection with PCV2 is necessary but not sufficient for pigs to develop PCVD. Infection with PCV2 alone does not generally cause overt clinical disease [2]. Most of the available data indicate that appropriate host, management, co-infection, immunostimulation, and nutrition are crucial for disease progression to PCVD [3]. However, the pathogenic mechanism of PCV2 remains poorly understood.

Glutamine (Gln) is the most abundant free amino acid in serum; this amino acid is important for the regulation of metabolism, cell integrity, protein synthesis, redox potential, gene expression, and intracellular signaling pathways [4]. Glutamine can be produced in sufficient quantities under normal physiological conditions, but becomes an essential amino acid during pathological or stress conditions [5]. Dietary l-glutamine supplementation can reportedly ameliorate the reproductive failure caused by PCV2 [6] and enhance the immune functions in PCV2-infected mice [7]. Dietary glutamine supplementation may confer a positive effect on the improvement of pregnancy outcomes in PCV2-infected mice by enhancing the immune response and the ability to clear PCV2 [6]. However, the mechanism by which glutamine affects PCV2 replication remains unclear.

Glutamine has an important role in cell culture in vitro. This amino acid is required by all mammalian and invertebrate cell lines as an energy substrate and a precursor for nucleotide, glutamate, and glutathione synthesis [8]. Furthermore, previous studies have suggested that glutamine affects the replication of viruses through different mechanisms within host cells. Dietary glutamine supplementation can protect the host from inflammation and infection [9] by stimulating glutathione synthesis in animal cells [10], which may lead to the activation of p38 mitogen-activated protein kinase (MAPK) [11,12]. Glutamine-deficient medium has been demonstrated to increase psittacosis virus multiplication [13], whereas Sendai virus proliferation in BHK cells is suppressed by a lack of glutamine [14]. In addition, glutamine deprivation enhances the plating efficiency of a herpes simplex virus type 1 ICP0-null mutant [15]. During human cytomegalovirus infection, glutamine is essential for virion production in cells [16]. Glutamine deficiency triggers the decrease in cellular glutathione (GSH) levels and promotes oxidative stress in HuH-7 cells [17]. In addition, GSH supplementation decreases DV2 production in HepG2 cells [18]. However, to the best of our knowledge, the influence of glutamine on PCV2 replication in PK-15 cells has not been reported to date.

Consequently, the present study investigated the effects of glutamine on PCV2 replication and its underlying mechanisms.

## Methods

### Cell culture and virus propagation

Dulbecco’s modified Eagle’s medium (DMEM) was used as the base medium for cell culture. The PCV1-free PK-15 continuous cell line was propagated and maintained at 37 °C in 5% CO_2_ in DMEM (Wisent, Nanjing, China) supplemented with 10% fetal calf serum, 100 U/mL penicillin, 0.1 mg/mL streptomycin, and 4 mM glutamine (hereafter referred to as complete medium) [19]. The wild-type PCV2 (PCV2NJ2002) used in the experiment was originally isolated from a kidney tissue sample of a pig with naturally occurring PMWS. The PCV type was determined by sequencing (TaKaRa Co., Dalian, China). PK-15 cells were infected with a PCV2 lysate at a multiplicity of infection (MOI) of 1. At 72 h after infection, approximately 26% of the PK-15 cells in the 96-well plates could be infected, as measured by indirect All experiments were undertaken following the guidelines of the Nanjing Agricultural University Ethics Committee, China.

### Reagents and antibodies

Rabbit antibodies specific for p38 and the phosphorylated form of p38 (p-p38) were obtained from Cell Signaling Technology. The porcine anti-PCV2 antibody for immunofluorescence was purchased from Univ Biotech Co. Ltd., Shanghai, China. The FITC-linked secondary antibody (rabbit anti-pig IgG) was purchased from Sigma, St. Louis, USA.

### Cell viability assay

PK-15 cells (2 × 10^4^ cells/well) were plated onto 96-well plates in the presence of glutamine (0, 0.1, 0.5, 1, 2, 4, 8, and 16 mM). After 24 h of incubation, the cells were subjected to colorimetric 3-(4,5-dimethylthiazol-2-yl)-2,5-diphenyltetrazolium bromide (MTT) assay (Sigma) according to the protocol of the manufacturer. The absorbance of each well was measured at 550 nm, with a reference wavelength of 690 nm.

### Quantification of PCV2 DNA by real-time PCR

The presence of newly synthesized viral DNA in the PK-15 cells was assayed by real-time PCR with the TaKaRa SYBR Green qPCR Kit (TaKaRa), as described elsewhere [20]. Briefly, the PCV2-infected PK-15 cells were harvested at 72 h post-inoculation, and DNA extraction was performed with a TaKaRa DNA Mini kit (TaKaRa). The purified DNA was used as a template for real-time PCR amplification. A 117 bp region was amplified from ORF2 of the PCV2 gene with a pair of PCV2-specific primers (forward primer: 5′-TAGTATTCAAAGGGCACAG-3′; reverse primer: 5′-AAGGCTACCACAGTCAG-3′). Quantitative real-time PCR was performed with the ABI PRISM 7300 Detection System (Applied Biosystems, Foster, USA). A recombinant pMD19 plasmid vector (TaKaRa) containing PCV2 genome inserts was used as the standard. Tenfold serial plasmid dilutions were tested and used to construct the standard curve by plotting the logarithm of the plasmid copy number against the measured CT values. The generated standard curve covered a linear range of 1 × 10^4^–1 × 10^10^ copies/mL for PCV2. Regression analysis of the linear portion of the curve produced a slope coefficient of −0.326, whereas the linear correlation (*R*^2^) between the logarithm of the plasmid copy number and CT was 0.998, with a *Y*-intercept of 45.087. The number of PCV2 DNA copies was calculated from the number of cells, specifically, the average amount of PCV2 DNA copies per 10^6^ cells, according to previous publications [21,22].

### Indirect immunofluorescence of PCV2-infected cells

At 72 h post-inoculation, the PK-15 cells were washed with phosphate-buffered saline (PBS) and fixed in methanol. The fixed cells were incubated in PBS containing 2% bovine serum albumin (BSA) at room temperature for 1 h, followed by incubation with the porcine anti-PCV2 antibody (Univ Biotech; diluted to 1:50 in PBS containing 1% BSA) at 37 °C for 1 h. After washing with PBS containing 0.1% Tween-20, cells were incubated with the FITC-conjugated rabbit anti-pig antibody (diluted to 1:100 in PBS containing 1% BSA) at 37 °C for 1 h. After washing in PBS, the cells were examined under a fluorescence microscope. Cells with the fluorescent stain (judged to be positive for the PCV2 viral antigen) were counted in six fields of view for each concentration in three replicated cultures. The calculation of the relative proportions of infected cells was based on the total amount of cells in each field.

### Western blot analysis

The cell lysates were suspended in RIPA lysis buffer (Beyotime, Haimen, China). PMSF was added to the cell suspensions, followed by centrifugation at 8000 × *g* for 10 min at 4 °C. The supernatant fluids were stored at −80 °C before use. For each sample, 50 μg was subjected to SDS-PAGE, and the separated proteins were transferred onto a polyvinylidene difluoride membrane (Bio-Rad, Hercules, USA). The membranes were blocked for 1 h at room temperature in Tris-buffered saline (TBS) containing 5% BSA and 0.1% Tween 20. Subsequently, the membranes were incubated with the respective specific primary antibodies against p-p38, p38, and β-actin at 4 °C overnight. The membranes were washed thrice with TTBS buffer and incubated with the respective secondary antibodies for 1 h at room temperature. The Western blots were visualized with the SuperSignal ECL kit (Thermo, Waltham, USA).

### SiRNA transfection

Double-stranded RNA sequences were designed with Invitrogen BlockiT RNAi Designer according to the *Sus scrofa* p38 mRNA sequence (Genbank accession number XM_003356616.1); the designed sequences were then synthesized by Invitrogen. The p38-specific siRNA sequence was 5′-GCAGGAGCUGAACAAGACATT-3′. The control siRNA had the following sequence: 5′-UUCUCCGAACGUGUCACGUTT-3′. The duplexes were resuspended in DEPC-treated water to obtain 20 μM solutions prior to use. PK-15 cells in DMEM with 10% fetal bovine serum (FBS) but without antibiotics were seeded into 24-well plates at a cell density of 5 × 10^4^ cells/well. The cells were incubated for 24 h at 37 °C in a 5% CO_2_ atmosphere. When the cells were 30%–50% confluent, siRNA was introduced via the X-tremeGene siRNA transfection reagent (Roche, Mannheim, Germany), according to the manufacturer’s protocol. Subsequently, 2.5 μL transfection reagent and 0.5 μg siRNA were added to each well, and the cells were incubated for 4 h. Finally, the cells were washed with DMEM and cultured for another 20 h in DMEM containing 2% FBS before the subsequent experiments.

### Determination of GSH and MDA in PK-15 cells

Cell extracts were prepared by sonication in ice-cold PBS. After sonication, the cell lysates were centrifuged at 10 000 × *g* for 20 min to remove the cell debris. The amount of reduced glutathione and maliondehyde (MDA) in the supernatants were measured with commercially available kits (Jiancheng Co., Nanjing, China), as previously described [23,24]. GSH was spectrophotometrically measured (412 nm) via a reaction with 5,5′-dithiobis(2-nitrobenzoic acid); the GSH concentrations were expressed as the micromoles of GSH per gram of protein. MDA was spectrophotometrically assayed at 548 nm via the thiobarbituric acid reaction; the MDA concentrations were expressed as micromoles per gram of protein. The total protein concentration was determined with a Bradford Protein Assay Kit (Beyotime). All samples were assayed in triplicate.

### Statistical analysis

The experimental data was analyzed by one-way analysis of variance (ANOVA). The least-significant difference test was used to compare the differences among the groups. The results are presented as the mean ± SD of replicates. Differences among groups were considered significant when *P* < 0.05.

## Results

### Glutamine promotes the viability of PK-15 cells

**Figure 1:**
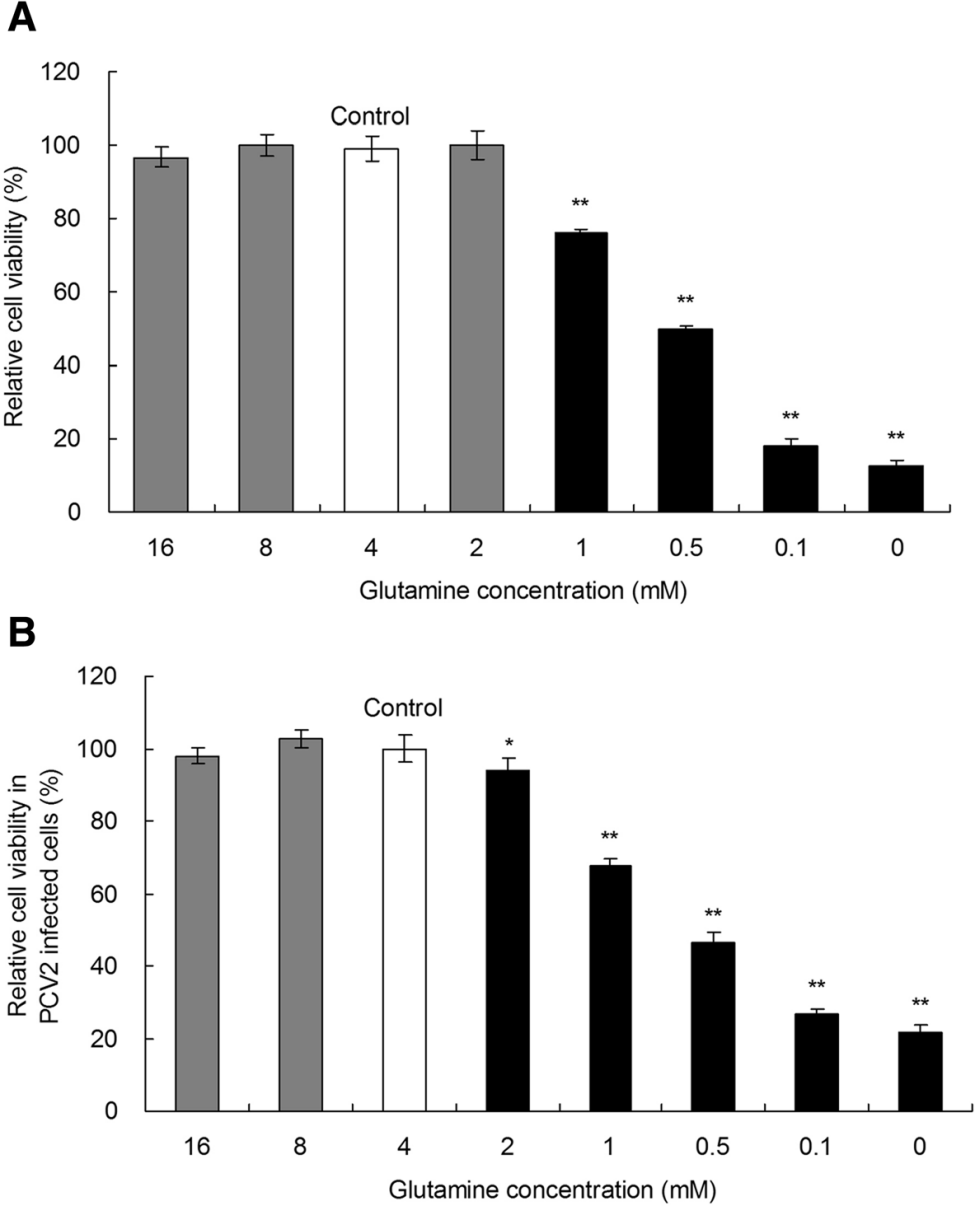
**Effects of glutamine on viability of PK-** **15 cells.** PK-15 cells without **(A)** or with **(B)** PCV2 infection were grown in 96-well plates with DMEM containing 4 mM glutamine for 24 h until 50% confluence was reached. Subsequently, the medium was changed to fresh DMEM with various concentrations of glutamine (0–16 mM). The cells were incubated for an additional 48 h before the cell viability was determined by MTT assay. Values are given as mean ± SD from three independent experiments. Groups were compared by a 1-way ANOVA followed by a least-significant difference test (*P < 0.05, **P < 0.01).

To assess the effect of glutamine on the growth of PK-15 cells with or without PCV2 infection, PK-15 cells were grown in 96-well plates with DMEM containing 4 mM glutamine for 24 h until 50% confluence was reached. Subsequently, the medium was changed to fresh DMEM with various concentrations of glutamine (0–16 mM). The cells were incubated for an additional 48 h before the cell viability was determined. As shown in Figures 1A and 1B, the significant promotion of cell viability was observed when glutamine was present at 4 mM or higher as compared with groups subjected to glutamine starvation (*P* < 0.05). The cell viability was significantly decreased by 24%, 50%, 81%, and 87% in non-infected groups incubated with glutamine at concentrations of 1, 0.5, 0.1, and 0 mM, respectively, as compared with the controls with 4 mM glutamine treatment. The relative cell viability of the infected cells was 93%, 68%, 49%, 28%, and 23% when glutamine was supplemented at concentrations of 2, 1, 0.5, 0.1, and 0 mM, respectively. The promotion of PK-15 cell viability by glutamine was concentration dependent within the range of 0–2 mM glutamine. Our data indicate that glutamine plays an important role in PK-15 cell viability. Glutamine starvation inhibited the growth of PK-15 cells when its concentration was less than 4 mM in the medium.

### Effect of glutamine starvation on PCV2 replication in PK-15 cells

**Figure 2:**
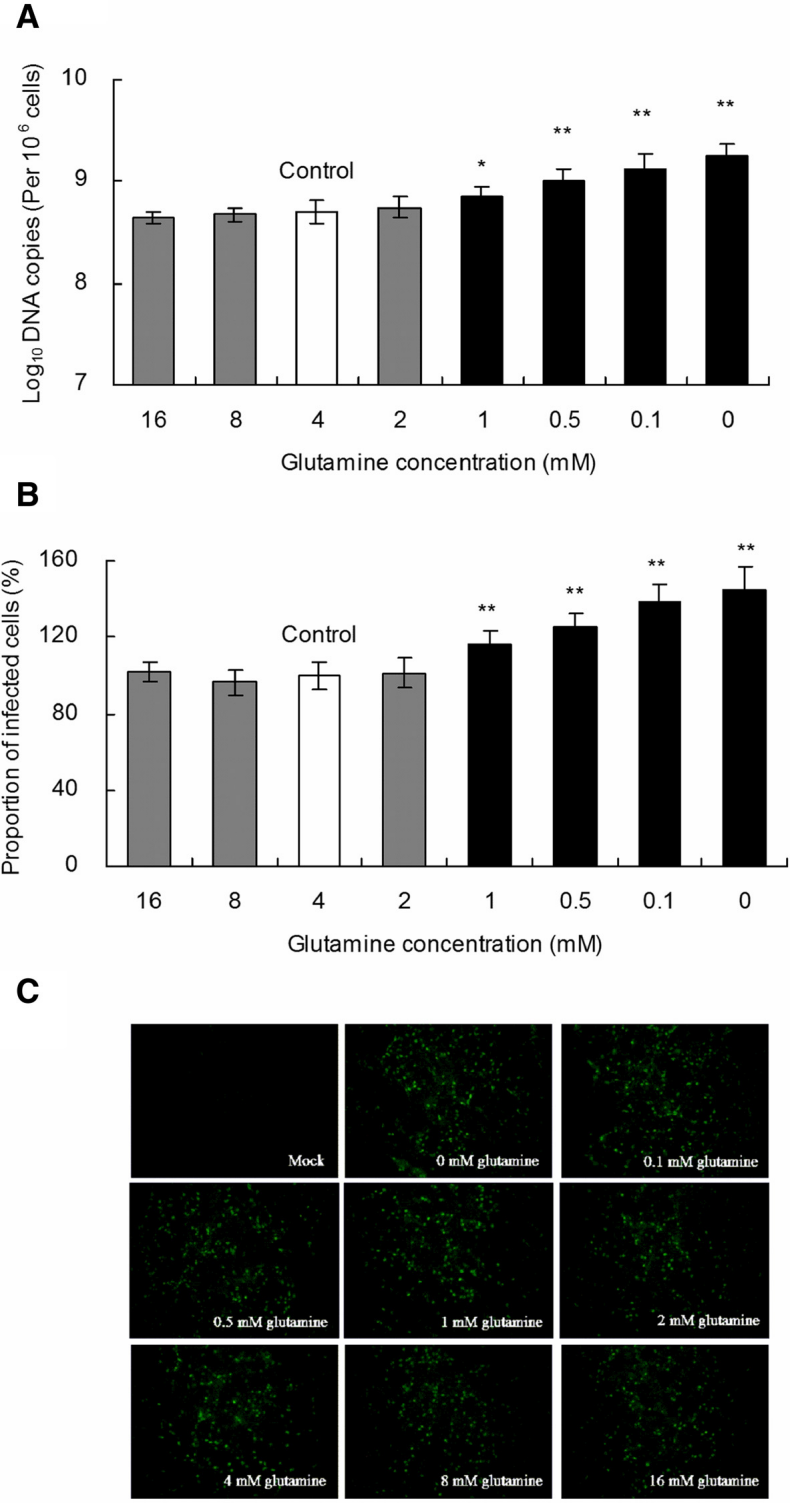
**Effects of different concentrations of glutamine on PCV2 replication.** PK-15 cells were infected with PCV2 at an MOI of 1 and incubated in the complete medium for 24 h. Subsequently, PK-15 cells were grown in medium containing various concentrations of glutamine. After 48 h, the PK-15 cells were harvested to determine the number of PCV2 *log*_10_ DNA copies per 10^6^ cells **(A)** and the relative proportion of PCV2-infected cells (**B** and **C**) by real-time PCR and immunofluorescence microscopy, respectively. Values shown are means ± SD mean from three independent experiments. Asterisks indicate groups statistically significantly different from control by a 1-way ANOVA followed by least-significant difference test (**P < 0.01).

To establish the viral infection and ensure that PK-15 cells were able to survive in glutamine-free medium in our experiment, the cells were infected with PCV2 at a MOI of 1 and incubated in the complete medium for 24 h. Subsequently, PK-15 cells were grown in media containing various concentrations of glutamine. After 48 h, the PK-15 cells were harvested to determine the number of PCV2 DNA copies and the relative proportion of PCV2-infected cells by real-time PCR and immunofluorescence microscopy, respectively. The PCV2 *log*_10_ DNA copies per 10^6^ cells (Figure 2A) and the proportion of PCV2 infected cells (Figures 2B and 2C) were significantly increased when the glutamine concentration was below 2 mM as compared with cells grown with >4 mM glutamine (*P* < 0.05). The number of PCV2 *log*_10_ DNA copies per 10^6^ cells was significantly increased by 2%, 4%, 5%, and 6% in groups with glutamine starvation at concentrations of 1, 0.5, 0.1, and 0 mM, respectively, as compared with the controls treated with 4 mM glutamine. The relative proportions of the PCV2-infected cells were 116%, 125%, 138%, and 145% when glutamine was supplemented at concentrations of 1, 0.5, 0.1, and 0 mM, respectively. Our results show that glutamine starvation increases PCV2 replication.

**Figure 3:**
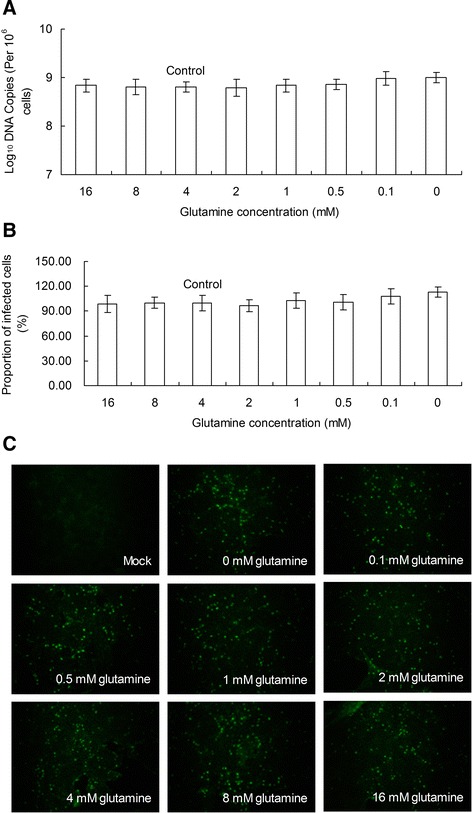
**Glutamine supplementation blocks the promotion of PCV2 replication by glutamine starvation.** PK-15 cells were infected with PCV2 at an MOI of 1 in complete medium for 24 h. These PK-15 cells were subsequently grown in medium containing various concentrations of glutamine for 48 h before the media of all treatment groups were changed to fresh media with 4 mM glutamine. The cells were incubated for another 48 h and then harvested to determine the number of PCV2 *log*_10_ DNA copies per 10^6^ cells **(A)** and the relative proportion of PCV2 infected cells (**B** and **C**). Groups were compared by a 1-way ANOVA followed by a least-significant difference test. Significant changes are indicated by *(*P* < 0.05) in comparison with control.

Another set of experiments was performed to determine whether glutamine supplementation blocks the positive effect of glutamine starvation on PCV2 replication. First, PK-15 cells were infected with PCV2 at an MOI of 1 in complete medium for 24 h. These PK-15 cells were subsequently grown in medium containing various concentrations of glutamine for 48 h before the media of all treatment groups were changed to fresh media with 4 mM glutamine and the cells were incubated for another 48 h. Finally, the number of PCV2 DNA copies and the relative proportion of PCV2-infected cells were calculated. As shown in Figure 3, the shift to fresh media with 4 mM glutamine at 72 h and the additional incubation for 48 h did not significantly alter the PCV2 replication in all groups (*P* > 0.05). The results indicate that the addition of fresh media with 4 mM glutamine blocked the promotion of PCV2 replication by glutamine starvation.

### GSH levels decrease as the MDA concentration increases in PK-15 cells with glutamine starvation

**Figure 4:**
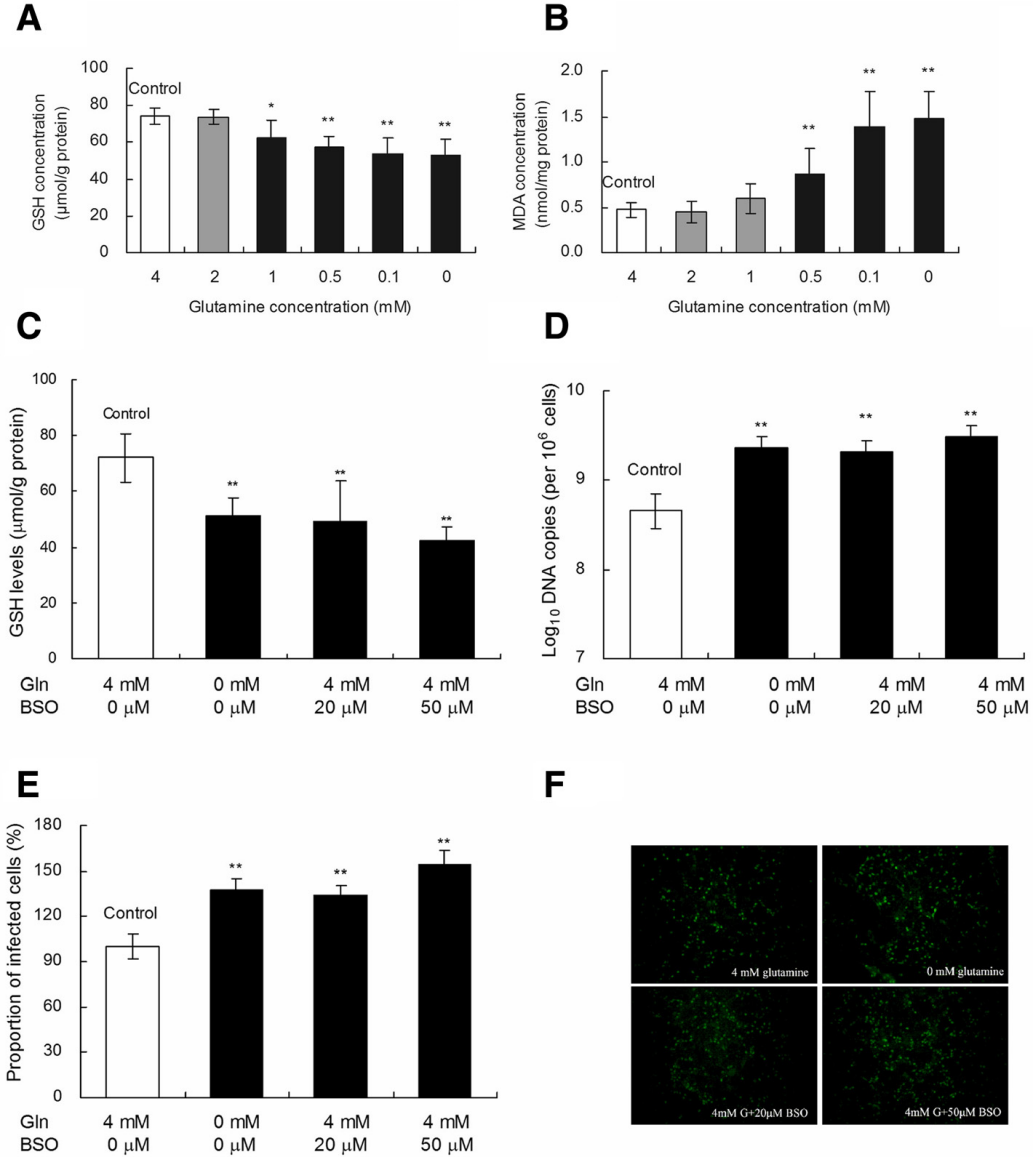
**Effect of glutamine starvation on GSH levels**, **MDA concentration and PCV2 replication.** After 24 h of PCV2 infection in complete medium, fresh glutamine-free medium with various concentrations of glutamine and BSO was added to replace the original medium. The cells were cultured for another 48 h before the determining of GSH levels (**A** and **C**), MDA concentration **(B)**, the number of PCV2 *log*_10_ DNA copies per 10^6^ cells **(D)**, and the proportion of PCV2 infected cells (**E** and **F**). Values are shown as mean ± SD from three independent experiments. Asterisks indicate groups statistically significantly different from control by a 1-way ANOVA followed by a least-significant difference test (*P < 0.05, **P < 0.01).

Glutamine can reportedly act as a precursor for glutathione synthesis [25]. Given the findings of previous studies [26,27], we hypothesized that the increased replication of PCV2 in PK-15 cells during glutamine starvation may be attributed to decreased GSH levels. To test this hypothesis, intracellular GSH levels were measured in PCV2-infected cells grown with various glutamine concentrations. After 24 h of PCV2 infection in complete medium, fresh glutamine-free medium with various glutamine concentrations was added to replace the original medium, and the cells were cultured for another 72 h. As shown in Figure 4, the intracellular GSH levels were significantly decreased by 16%, 23%, 28%, and 29% in PK-15 cells with glutamine starvation at concentrations of 1, 0.5, 0.1, and 0 mM, respectively, as compared with the control cells with 4 mM glutamine (Figure 4A; *P* < 0.05). The MDA concentration was significantly increased by 82%, 191%, and 211% in PK-15 cells with glutamine starvation at concentrations of 0.5, 0.1, and 0 mM, respectively (Figure 4B; *P* < 0.01). These results suggest that glutamine starvation induced oxidative stress by decreasing the GSH level and increasing the MDA concentration in PK-15 cells.

### PCV2 replication increases in PK-15 cells after GSH synthesis is inhibited by BSO

To further confirm the role of decreased GSH levels in the promotion of PCV2 replication induced by glutamine starvation, BSO was used; this compound is a well-known specific inhibitor of GSH synthesis [28]. After 24 h of PCV2 infection, the complete medium was removed, and the infected cells were cultured for another 48 h in the absence of glutamine or in the presence of glutamine and BSO. Compared with the control group grown with 4 mM glutamine, the intracellular GSH concentrations were significantly reduced in the PK-15 cells treated with glutamine and BSO (*P* < 0.01; Figure 4C). Therefore, exogenous BSO attenuated the intracellular GSH levels in the presence of glutamine. The number of PCV2 DNA copies were increased (Figure 4D) and the relative proportion of infected cells (Figures 4E and 4 F) were enhanced by 36.2% and 51.0% in the presence of 4 mM glutamine after treatment with 20 and 50 μM BSO, respectively, as compared with the controls (*P* < 0.01). These results showed that PCV2 replication was increased in PK-15 cells after GSH synthesis was inhibited by BSO. Therefore, GSH may play an important role in the promotion of PCV2 replication during glutamine starvation.

### Glutamine starvation or BSO treatment increase the level of p38 MAPK phosphorylation

**Figure 5:**
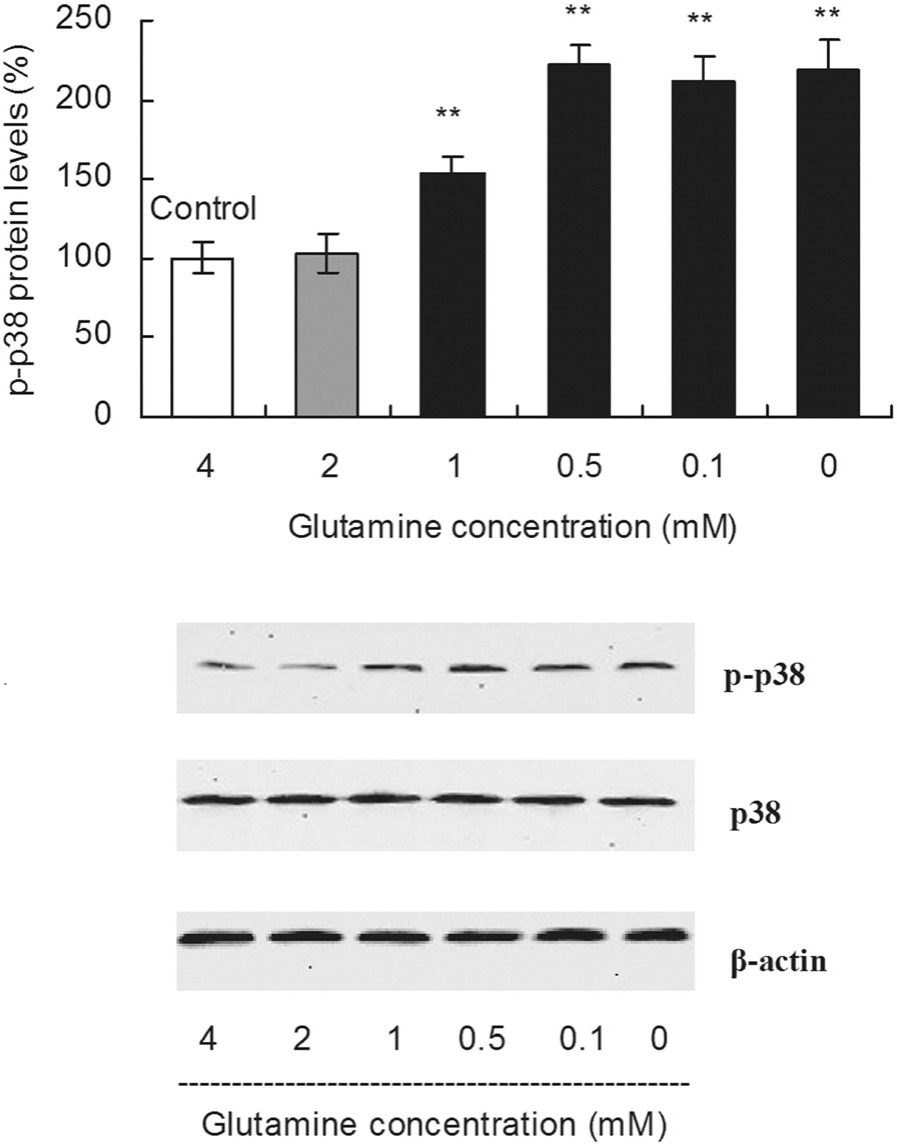
**Glutamine starvation increases the level of p38 MAPK phosphorylation.** After 24 h of PCV2 infection, the complete medium was removed, and fresh medium with various concentrations of glutamine was added for an additional 48 h of incubation. The protein levels of p38 phosphorylation were measured by Western blot. Values are shown as mean ± SD from three independent experiments. Asterisks indicate groups statistically significantly different from control by a 1-way ANOVA followed by a least-significant difference test (**P < 0.01).

p38 MAPK has been confirmed to have an important role in the efficient replication of PCV2 in PK-15 cells [29]. Therefore, the effect of glutamine starvation on PCV2 replication may be associated with the phosphorylation of p38 MAPK, which is induced by attenuated GSH synthesis. To address this question, we calculated the rate of p38 MAPK phosphorylation in PK-15 cells by measuring the p38 mRNA and protein levels via real-time PCR and Western blot analysis, respectively. After 24 h of PCV2 infection, the complete medium was removed, and fresh medium with various concentrations of glutamine was added for an additional 48 h of incubation. As shown in Figure 5, the levels of p38 MAPK phosphorylation increased by 1.53-, 2.21-, 2.12-, and 2.19-fold after supplementation with 1, 0.5, 0.1 and 0 mM glutamine, respectively, as compared with the controls incubated with 4 mM glutamine (Figure 5; *P* < 0.01). However, the relative p38 mRNA levels were not significantly different among groups with various concentrations of glutamine (data not shown). These results indicate that glutamine starvation increased the level of p38 MAPK phosphorylation.

**Figure 6:**
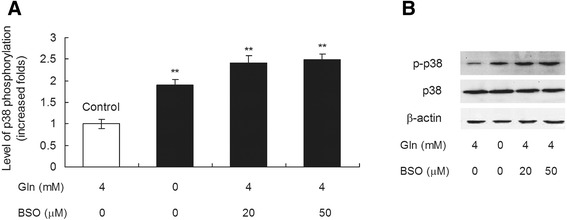
**Glutamine starvation increases the phosphorylation of p38 MAPK via the reduced GSH synthesis.** PK-15 cells lysates were extracted to measure the protein levels of p38 phosphorylation by Western blot after incubation for 72 h in the absence of glutamine or in the presence of glutamine and BSO **(A** and **B)**. Values are shown as mean ± SD from three independent experiments. Asterisks indicate groups statistically significantly different from control by a 1-way ANOVA followed by least-significant difference test (**P < 0.01).

To further confirm whether the phosphorylation of p38 MAPK was induced by glutamine starvation via the reduced GSH synthesis, PK-15 cell lysates were extracted to measure the rate of p38 phosphorylation after incubation for 72 h in the absence of glutamine or in the presence of glutamine and BSO. As shown in Figure 6, glutamine starvation increased the levels of p38 MAPK phosphorylation by 2.07-fold as compared with the controls treated with 4 mM glutamine (*P* < 0.01). In the presence of 4 mM glutamine, the levels of p38 MAPK phosphorylation in the groups with 20 or 50 μM BSO treatment increased by 2.42- and 2.50-fold, respectively, as compared with the controls (*P* < 0.01). The total p38 protein levels were not significantly different among all groups (*P* > 0.05). These results suggest that glutamine starvation and BSO treatment increased the level of p38 MAPK phosphorylation.

### Knockdown of p38 MAPK decreased PCV2 replication but did not affect the level of GSH and MDA in PK-15 cells

**Figure 7:**
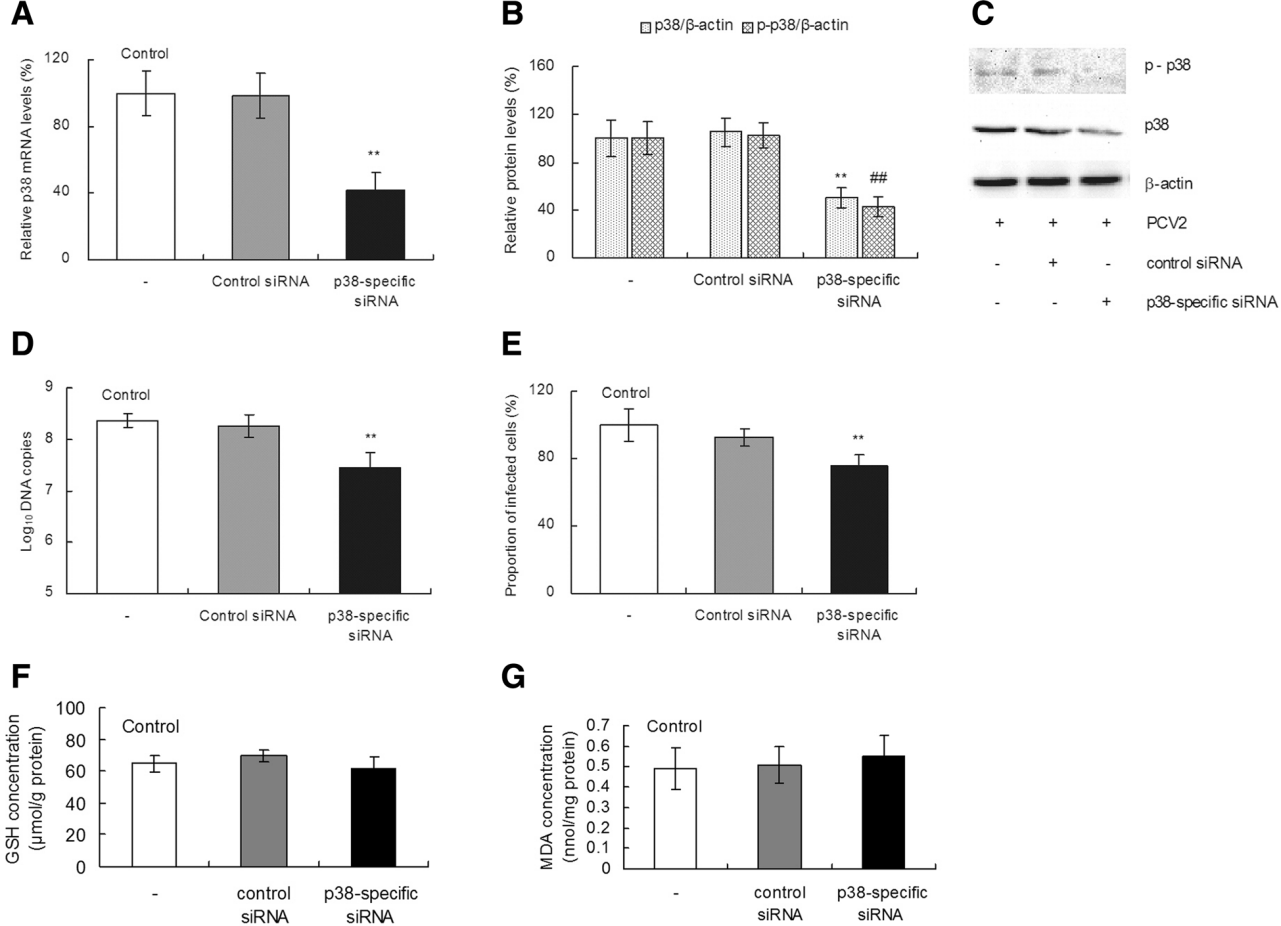
**Knockdown of p38 MAPK decreases PCV2 replication but does not affect the level of GSH and MDA in PK-15 cells.** PCV2-infected cells were transfected with p38-specific or control siRNA. After 5 h of transfection treatment, the medium was removed, and fresh basal medium was added. After transfected-cells were cultured for another 72 h, the relative mRNA levels of p38 **(A)**, the relative proteins levels of p38 and p-p38 (**B** and **C**), the number of PCV2 *log*_10_ DNA copies per 10^6^ cells **(D)**, the relative proportion of infected cells **(E)**, the level of GSH **(F)** and MDA **(G)** were assayed as described in Methods. Values are given as mean ± SD from three independent experiments. Groups were compared by a 1-way ANOVA followed by least-significant difference test. Significant changes are indicated by **(*P* < 0.01) and ## (*P* < 0.01) in comparison with controls.

The extent of p38 MAPK knockdown was evaluated by determining the relative p38 mRNA and protein levels after infected PK-15 cells were transfected with p38-specific or control siRNA. As shown in Figure 7, the transfection of p38-specific siRNA into PK-15 cells decreased the relative p38 mRNA and protein levels as compared with the controls. The relative p38 mRNA, p38 protein, and p-p38 protein levels were decreased by 57%, 50%, and 58%, after transfection (Figures 7A, 7B, and 7C; *P* < 0.01). Transfection of PK-15 cells with anti-p38 siRNA decreased PCV2 replication as measured by the number of PCV2 DNA copies and the proportion of infected cells. A significant decrease of approximately 11% in the PCV2 *log*_10_ DNA copies per 10^6^ cells (Figure 7D) and 25% in the proportion of infected cells (Figure 7E) was observed in p38-knockdown cells (*P* < 0.01). As shown in Figures 7F and 7G, the knockdown of p38 had no effect on the intracellular GSH levels and the MDA concentration (*P* > 0.05). Our results demonstrate that p38 MAPK knockdown decreases PCV2 replication.

### PCV2 replication promoted by glutamine starvation could be blocked by p38 knockdown in PK-15 cells

**Figure 8:**
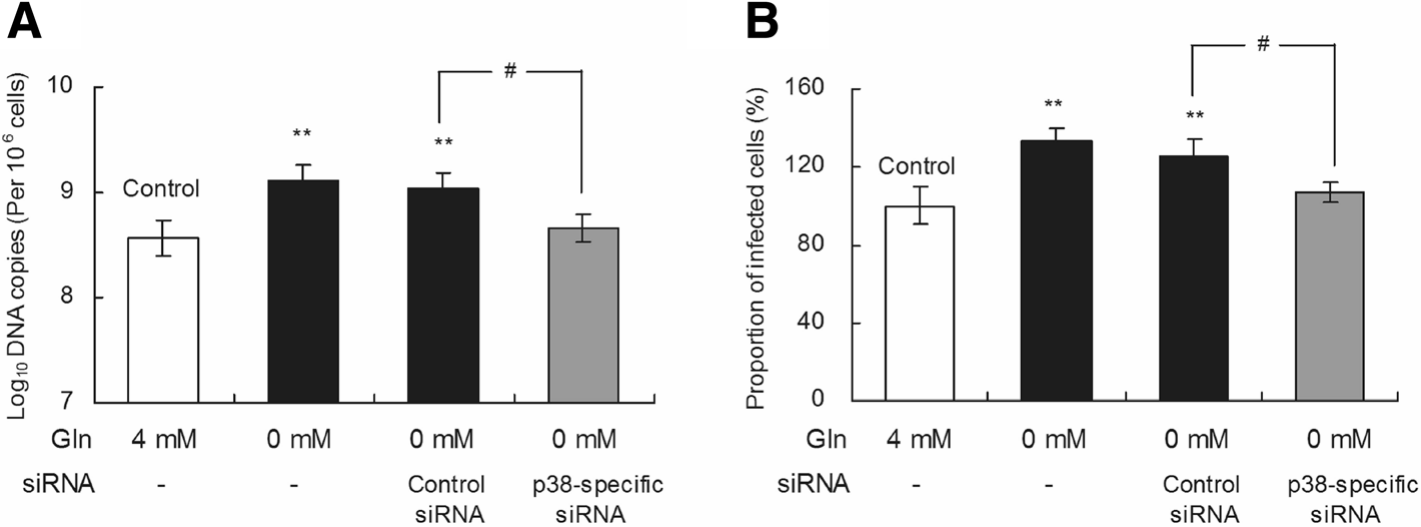
**PCV2 replication promoted by glutamine starvation could be blocked by p38 knockdown in PK**-**15 cells.** PCV2-infected cells were transfected with p38-specific, control or non siRNA. After 5 h of transfection treatment, the medium was removed, and fresh basal medium with various concentrations of glutamine was added. After transfected-cells were cultured for another 72 h, the number of PCV2 *log*_10_ DNA copies per 10^6^ cells **(A)** and the relative proportion of infected cells **(B)** were assayed by real-time PCR and immunofluorescence microscopy, respectively. Values are given as mean ± SD from three independent experiments. Groups were compared by a 1-way ANOVA followed by least-significant difference test. Significant changes are indicated by **(*P* < 0.01) and # (*P* < 0.05) in comparison with controls.

The influence of glutamine starvation on PCV2 replication in p38-knockdown cells was evaluated to further investigate the role of p38 in the promotion of PCV2 replication by glutamine starvation. A significant increase of approximately 6% and 5% in the number of PCV2 *log*_10_ DNA copies per 10^6^ cells as well as approximately 33% and 25% in the proportion of infected cells in the groups treated with non-siRNA and control siRNA (*P* < 0.01; Figure 8A), respectively, but no increase was detected in groups treated with p38-specific siRNA (*P* > 0.05), as compared with the controls without glutamine starvation. Between the groups with glutamine starvation and siRNA treatment, the number of PCV2 *log*_10_ DNA copies per 10^6^ cells and the proportion of infected cells in groups treated with p38-specific siRNA decreased to normal levels as compared with groups treated with control siRNA. These results show that the promotion of PCV2 replication caused by glutamine starvation could be blocked in p38-knockdown cells.

## Discussion

PCV2 infects pigs worldwide; this virus has been linked to several pathological conditions collectively named PCVD [30-32]. Not all pigs infected with PCV2 develop PCVD, although PCV2 is recognized as an essential infectious agent of PCVD [3]. The infection of pigs with PCV2 and other unknown triggers are required for PCVD to occur [3]. The evaluation of the role of these triggers is essential to understand the observed incidence of PCVD. Dietary glutamine supplementation may have an inhibitory effect on PCV2 infection [6,7]. The initial purpose of this study was to investigate the effects of glutamine supplementation on PCV2 replication and the underlying mechanisms in vitro. However, glutamine supplementation at concentrations of 4–16 mM did not affect PCV2 replication in PK-15 cells. Surprisingly, glutamine starvation significantly increased PCV2 replication (Figure 2; *P* < 0.05). Further studies targeting the effect of glutamine starvation on PCV2 replication in vitro and in vivo may improve the treatment of PCVD and the production of PCV2 vaccines.

Previous studies investigated the effect of glutamine on virus growth. Human cytomegalovirus-infected cells failed to produce viruses under glutamine deprivation [16]. Likewise, the growth of HVJ (Sendai virus) was significantly suppressed in glutamine-starved BHK cells, but the VSV and NDV viruses were not markedly affected by glutamine starvation [14]. In the present study, the effect of glutamine on PCV2 replication in PK-15 cells differed from that of the above mentioned viruses. Glutamine starvation induced the significant increase of PCV2 (*P* < 0.05), but the addition of fresh media with 4 mM glutamine blocked the PCV2 replication promoted by glutamine starvation. The results of the present study were consistent with our previous study, wherein the reduction of GSH levels and the induction of oxidative stress both increased PCV2 replication in PK-15 cells [26,27,33].

Glutamine has an essential role in promoting and maintaining cell growth and function in rapidly dividing cells because this amino acid is an important precursor of proteins, amino sugars, purines, and pyrimidines [34]. Glutamine metabolism is crucial to produce ATP and glutamate, which are necessary for glutathione synthesis [35]. Glutathione is a tripeptide that contains glutamate, cysteine, and glycine; this particularly important antioxidant protects cells from free radical injury [36]. In Jurkat T cells, glutamine increases intracellular GSH and decreases oxidative stress [37]. Glutamine deficiency triggers the decrease of cellular GSH levels and the promotion of oxidative stress in HuH-7 cells [17]. In addition, GSH supplementation decreases DV2 production in HepG2 cells [18]. In the present study, glutamine deprivation decreased the GSH levels but increased the MDA concentration and PCV2 replication (*P* < 0.05; Figure 4). Once GSH synthesis is inhibited by BSO, there is also a significant increase of PCV2 replication in PK-15 cells without glutamine starvation (Figure 4; *P* < 0.01). These results indicate that glutamine starvation may increase PCV2 replication by reducing intracellular GSH levels in PK-15 cells.

The activation of p38 MAPK is essential for efficient PCV2 replication [29]. Intracellular GSH has a regulatory role in p38 MAPK activation [11,12]. A complex relationship may exist between glutamine starvation, GSH levels, p38 MAPK activation, and PCV2 replication. We demonstrate that glutamine starvation and BSO treatment did not change the level of p38 mRNA (data not shown). However, the lack of glutamine caused the reduced GSH levels, increased p38 MAPK phosphorylation, and increased PCV2 replication in PK-15 cells (Figures 4 and 5). p38 MAPK phosphorylation and PCV2 replication was significantly decreased by p38 knockdown in PK-15 cells without glutamine starvation (*P* < 0.01). Moreover, the promotion of PCV2 replication by glutamine starvation could be blocked by p38 knockdown in PK-15 cells (Figures 7 and 8). These results indicate that glutamine starvation increased PCV2 replication through intracellular GSH downregulation, which was associated with the promotion of p38 MAPK activation.

Recent studies have revealed that glutamine starvation causes the activation of p38 MAPK and the deactivation of mTOR, which then induces autophagy [38-40]. Moreover, PCV2 induces autophagy by repressing mTOR in a cascade of phosphorylated proteins involving TSC2, ERK1/2, and AMPK [41] and enhances the replication of autophagic machinery in PK-15 cells [42]. Therefore, the role of autophagy in PCV2 replication during glutamine starvation is an interesting topic for future studies. The results obtained in the present study improve the understanding of PCV2 pathogenesis.

In the present study, glutamine starvation affected PK-15 cell survival. To elucidate the exact mechanism by which glutamine starvation influences PCV2 replication, a different cell line would be preferred, whose survival is not affected by glutamine starvation. However, only a few available cell lines are permissive to PCV2 infection. Additionally, glutamine starvation affects PK-15 cells and other cell lines because glutamine is required for the growth and survival of almost all cells [43,44]. A cell line that is not affected by glutamine starvation is difficult to find. Therefore, we believe that a cell line that is suitable for PCV2 infection but not affected by glutamine starvation would be impossible to find. The present results are reasonable because previous studies have reported that glutamine supplementation can decrease PCV2 infection [6,7]. In addition, glutamine starvation can induce oxidative stress [25,35], whereas oxidative stress can improve PCV2 replication [26,27,33].

In conclusion, our results show that PCV2 replication increases under conditions of lower glutamine concentration (less than 4 mM). Glutamine starvation enhances PCV2 replication in PK-15 cells via the phosphorylation of p38 MAPK, which is related to the reduction of intracellular GSH levels. Our findings contribute towards the interpretation of the possible pathogenic mechanism of PCV2 and provide a theoretical reference for the application of glutamine in the control of PCVD.
